# Non-Targeted HPLC-UV Fingerprinting as Chemical Descriptors for the Classification and Authentication of Nuts by Multivariate Chemometric Methods

**DOI:** 10.3390/s19061388

**Published:** 2019-03-21

**Authors:** Guillem Campmajó, Gemma J. Navarro, Nerea Núñez, Lluís Puignou, Javier Saurina, Oscar Núñez

**Affiliations:** 1 Department of Chemical Engineering and Analytical Chemistry, University of Barcelona, Martí i Franquès, 1-11, E08028 Barcelona, Spain; campma03@gmail.com (G.C.); g.navarro.albiol@gmail.com (G.J.N.); nereant7@gmail.com (N.N.); lluis.puignou@ub.edu (L.P.); xavi.saurina@ub.edu (J.S.); 2 Research Institute in Food Nutrition and Food Safety, University of Barcelona, Recinte Torribera, Av. Prat de la Riba 171, Edifici de Recerca (Gaudí), Santa Coloma de Gramenet, E08921 Barcelona, Spain; 3 Serra Húnter Fellow, Generalitat de Catalunya, Rambla de Catalunya 19-21, E08007 Barcelona, Spain

**Keywords:** HPLC-UV, fingerprinting, food authentication, nuts, principal component analysis, partial least squares-discriminant analysis

## Abstract

Recently, the authenticity of food products has become a great social concern. Considering the complexity of the food chain and that many players are involved between production and consumption; food adulteration practices are rising as it is easy to conduct fraud without being detected. This is the case for nut fruit processed products, such as almond flours, that can be adulterated with cheaper nuts (hazelnuts or peanuts), giving rise to not only economic fraud but also important effects on human health. Non-targeted HPLC-UV chromatographic fingerprints were evaluated as chemical descriptors to achieve nut sample characterization and classification using multivariate chemometric methods. Nut samples were extracted by sonication and centrifugation, and defatted with hexane; extracting procedure and conditions were optimized to maximize the generation of enough discriminant features. The obtained HPLC-UV chromatographic fingerprints were then analyzed by means of principal component analysis (PCA) and partial least squares-discriminant analysis (PLS-DA) to carry out the classification of nut samples. The proposed methodology allowed the classification of samples not only according to the type of nut but also based on the nut thermal treatment employed (natural, fried or toasted products).

## 1. Introduction

Nowadays, food manufacturers, researchers, and society, in general, have become very interested in the quality of food products, not only from the nutritional point of view but also in relation to food safety issues or regarding the presence of bioactive substances with beneficial properties for consumers (functional foods, nutraceuticals, etc.). Within this context and considering the complexity of the food chain in a globalized world where many players are involved between production and consumption, food manipulation and adulteration practices are rising because of the ease of perpetrating fraud that may remain undetected. In general, food adulteration is carried out to increase volume, to mask the presence of inferior quality components, and to replace the authentic substances for the seller’s economic gain. For instance, a common fraud is the employment of a cheaper similar ingredient, which the consumer has difficulty recognizing and which is difficult to detect by current analytical methodologies. However, it must be considered that the deliberate adulteration of food and its misrepresentation to deceive final consumers is illegal worldwide [1]. In addition, depending on the nature of the adulterants, the fraudulent food product can also represent a health risk for the consumer when prohibited substances are added to deceive its organoleptic properties or when the adulterant can produce allergy episodes. Sixty-eight percent of food fraud violations are perpetrated in animal and vegetable products with high-fat content becoming a crucial issue for food processing industries [2]. Therefore, the ability to guarantee food integrity and authenticity is a major concern in the food industry for both economic and safety reasons, requiring new analytical methodologies.

Nuts are food products with important health benefits to humans. Even though approximately 70% of their weight is attributed to fat, the amount of saturated fatty acids is very low. Thus, their high unsaturated fatty acid content induces the reduction of both total and low-density lipoprotein cholesterols [3], correlating their consumption to the decrease of ischemic heart disease [4]. They have also been shown to be important sources of antioxidant compounds, such as polyphenols [5,6,7,8], which are secondary metabolites and the largest group of phytochemicals in plants. In fact, the main dietary sources of total polyphenols are nuts, followed by tea and coffee, rich in flavanols and hydroxycinnamic acids, respectively [9]. Walnuts, almonds, and hazelnuts are the most commonly consumed nuts in the European countries (either raw, fried or toasted) where tree nuts are more consumed than peanuts or seeds. Furthermore, walnuts and almonds contain high levels of total polyphenols in comparison to other polyphenol-rich foods, such as apple juice or red wine [8].

Nuts should be considered as highly exposed to fraud practices since they can be relabeled with old or expired stock samples or replaced with cheaper ones, representing serious problems to consumers with allergies and intolerances [10]. Of the 177 cases of fraudulent practices reported in the European Union in 2016, 4% were related to nuts and seeds [2]. Several analytical methodologies in combination with chemometrics have been described in the literature to address nut authentication and to detect its fraud. For example, the determination of fatty acids by gas chromatography-flame ionization detector (GC-FID) in combination with principal component analysis (PCA) was proposed for the authentication of almond cultivars [11], for the classification and authentication of Iranian walnuts according to their geographical origin [12], and to authenticate several almond genotypes grown in Serbia [13]. In this last work, fatty acid content was combined with the determination of some phenolic compounds by ultra-high performance liquid chromatography-tandem mass spectrometry (UHPLC-MS/MS). The fatty acid profile obtained by GC-FID and analyzed by PCA, linear discriminant analysis (LDA), and partial least squares (PLS) regression was also proposed to detect and quantify the fraudulent addition of apricot kernel in almonds [14]. However, time-consuming sample treatments are typically needed for fatty acid determination by GC techniques, which require derivatization steps to obtain volatile fatty acid methyl esters. Thus, spectroscopic techniques, such as near-infrared (NIR), have been employed for the classification of hazelnuts according to protected designations of origin (PDO) [15] or to address the discrimination of peanuts from bulk cereals and nuts [16]. Multi-elemental analysis fingerprinting based on inductively coupled plasma optical emission measurements (ICP-OES) by determining 10 metallic elements and their analysis by PCA, LDA, and PLS were also described in adulteration studies of almond powder samples with peanut [10].

Most of the previously described methods are based on targeted approaches in which a specific group of known selected chemicals or belonging to the same family is determined. Generally, as the concentration (or peak signal) of these targeted compounds is used as a food feature (marker) to address food integrity and authenticity, this approach requires a previous quantification step using standards for each one. However, when dealing with food products, which are very complex matrices, the quantification of some of these chemicals may be a difficult task, especially due to the possibility of unknown interfering compounds. Hence, nowadays the use of non-targeted fingerprinting approaches, in which analytical signals related to the composition of foodstuffs are employed in a non-selective way (i.e., spectrum or chromatogram), are gaining popularity in food authentication [17,18,19,20,21]. Mathematical processing of the information in such fingerprints may allow the characterization and/or authentication of foodstuffs.

In this work, a non-targeted high-performance liquid chromatography with ultraviolet detection (HPLC-UV) fingerprinting method has been evaluated for the classification and authentication of different types of nuts. For that purpose, a total of 149 nut samples belonging to different classes (almonds, cashew nuts, hazelnuts, macadamia nuts, peanuts, pinions, pistachios, pumpkin seeds, sunflower seeds, and walnuts), some of them in different presentation formats according to the thermal treatment applied (natural, fried or toasted), were analyzed. Samples were extracted by a simple solid–liquid extraction method and the extracting solvent composition was optimized to maximize the total amount of non-targeted components extracted. Data corresponding to the non-targeted HPLC-UV fingerprints recorded at 280 nm were considered as a source of potential descriptors to be exploited for the characterization and classification of the analyzed nut samples by exploratory PCA and supervised partial least squares-discriminant analysis (PLS-DA).

## 2. Materials and Methods

### 2.1. Chemicals and Standard Solutions

All the reagents employed, unless otherwise stated, were of analytical grade. Methanol and acetonitrile (both UHPLC-gradient grade) were purchased from Panreac (Barcelona, Spain). Acetone, hexane, and formic acid (96%) were obtained from Sigma-Aldrich (St. Louis, MO, USA) and absolute ethanol from VWR International Eurolab S.L. (Barcelona, Spain). Water was purified using an Elix 3 coupled to a Milli-Q system (Millipore, Bedford, MA, USA) and filtered through a 0.22 µm nylon membrane integrated into the Milli-Q system.

### 2.2. Instrumentation

An Agilent 1100 Series HPLC instrument was used to obtain the HPLC-UV chromatograms employed as the data in the chemometric methods. The instrument was equipped with a quaternary pump (G1311A), a degasser (G1322A), an autosampler (G1329A), a diode-array detector (G1315B) and a computer with the Agilent Chemstation software, all of them from Agilent Technologies (Waldbronn, Germany). HPLC-UV runs were obtained in reversed-phase mode by employing a porous-shell Kinetex C18 column (1000 mm × 4.6 mm I.D., 2.6 µm particle size) from Phenomenex (Torrance, CA, USA) at room temperature. Gradient elution mode using 0.1% (v/v) formic acid aqueous solution (solvent A) and methanol (solvent B) as mobile phase components was applied as follows: 0–30 min, linear gradient from 5 to 75% B; 30–32.5 min, linear gradient from 75 to 95% B; 32.5–35 min, isocratic step at 95% B; 35–35.1 min, back to initial conditions at 5% B; and from 35.1–40 min, at 5% B for column re-equilibration. A mobile phase flow rate of 0.4 mL/min and an injection volume of 5 µL were applied. HPLC-UV chromatographic fingerprints registered at 280 nm were employed.

### 2.3. Samples and Sample Treatment

A total of 149 nut samples, obtained from Barcelona markets, were analyzed. Table 1 shows the description, including the abbreviation used in this manuscript, the number of samples regarding the sample treatment applied (natural, fried or toasted products) and the sample origin.

Sample extraction was performed as follows: 0.125 g of crushed and homogenized nut sample were weighed into a 15 mL polypropylene tube and extracted with 3 mL of acetone:water (70:30 *v*/*v*) solution by stirring in a Vortex (Stuart, Stone, United Kingdom) for 1 min and by sonication for 15 min (5510 Branson ultrasonic bath, Hampton, NH, USA). The solutions were then centrifuged for 30 min at 3400 rpm (Rotanta 460 RS Centrifuge, Hettich, Germany). The supernatant extract was transferred to a new 15 mL polypropylene tube, defatted with 3 mL hexane by stirring in a Vortex for 1 min and centrifuged again for 15 min at 3400 rpm. Finally, the sample extract was filtered through 0.22 µm nylon filter (Scharlab, Sentmenat, Barcelona, Spain) and stored at −18 °C in 2 mL glass injection vial until HPLC-UV analysis.

In addition, a quality control (QC) was prepared by mixing 50 µL of each nut sample extract. This QC was employed to evaluate the repeatability of the method and the robustness of the chemometric results.

Samples were randomly analyzed with the proposed HPLC-UV method. A QC and a blank of water were injected at the beginning of the sequence and every 10 sample injections.

### 2.4. Data Analysis

SOLO chemometric software from Eigenvector Research was used for calculations with PCA and PLS-DA [22]. A detailed description of the theoretical background of these methods is given elsewhere [23].

The X-data matrices to be treated by PCA and PLS-DA consisted of the HPLC-UV chromatographic fingerprints obtained at 280 nm (absorbance intensities at different retention times). HPLC-UV chromatograms were pretreated to improve the data quality while minimizing solvent and matrix interferences, peak shifting, and baseline drifts. For additional details see reference [24]. The Y-data matrix in PLS-DA consisted of the sample class. Scatter plots of scores and loadings of the principal components (PCs), in PCA, and latent variables (LVs), in PLS-DA, were used to investigate the structure of maps of samples and variables, respectively. The plots of scores show the distribution of the samples revealing patterns that can be correlated to sample characteristics. The plots of loadings provide information on the most descriptive features contributing to sample discrimination.

## 3. Results and Discussion

### 3.1. Sample Treatment: Optimization of the Extracting Solvent Composition

**Figure 1:**
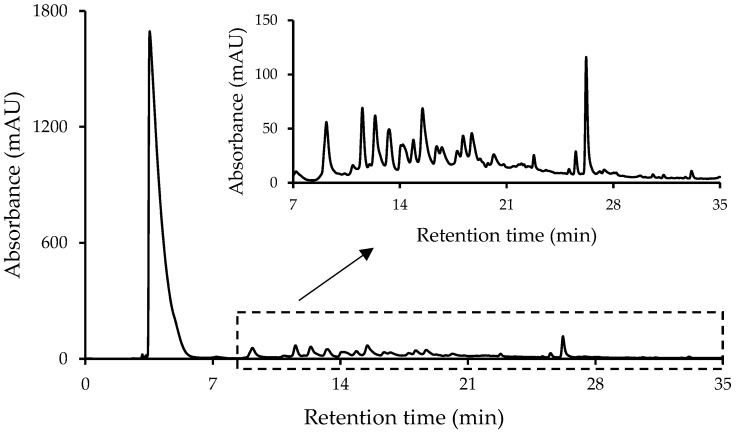
Non-targeted high-performance liquid chromatography with ultraviolet detection (HPLC-UV) chromatographic fingerprint at 280 nm of a walnut sample extracted with acetone:water 70:30 (*v*/*v*).

In the present work, a simple solid–liquid extraction procedure by stirring and sonication, followed by a defatting step with hexane, was proposed for the extraction of non-targeted phytochemicals from nut samples. An optimization of the extracting solvent composition was performed to maximize the total amount of extracted compounds. Thus, seventeen sample extraction conditions were evaluated for three nut samples (almond, hazelnut, and walnut), as they were extracted with different solvent mixtures including pure water, ethanol, methanol, acetonitrile, and acetone, as well as with organic solvent:water mixtures in 50:50, 70:30, and 80:20 (*v*/*v*) ratios. Considering the high polyphenolic content of nuts, a reversed-phase HPLC-UV method, previously developed for the determination of polyphenols in wine samples [25], was employed with some modifications by using a porous-shell C18 column and a gradient elution with 0.1% formic acid aqueous solution and methanol as mobile phase components (conditions described in Section 2.2). As an example, Figure 1 shows the non-targeted HPLC-UV chromatographic fingerprint obtained for a walnut sample extracted with acetone:water 70:30 (*v*/*v*).

**Figure 2:**
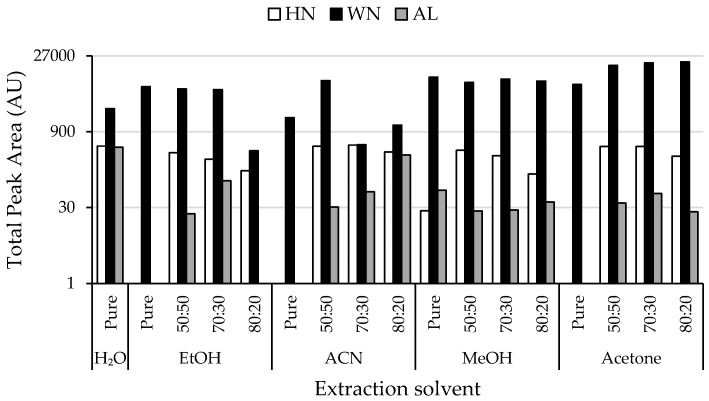
Total area of extracted phytochemical components (represented in logarithmic scale) obtained from HPLC-UV chromatographic fingerprints of a hazelnut (HN), walnut (WN) and almond (AL) sample extracted using different extraction solvent compositions. Ratios indicated correspond to organic solvent:water (*v*/*v*).

As seen in the figure, a high signal caused by the absorption of the acetone present in the extracting solvent was detected close to the dead volume (4–5 min). Moreover, a significant number of peak signals related to the non-targeted extracted compounds were obtained. In fact, to select the best extracting solvent, the total peak area (sum of each detected compound peak area without considering the one related to the organic solvent) was employed as a chemical parameter and the obtained non-targeted HPLC-UV chromatographic fingerprints were evaluated. Thus, Figure 2 shows the resulting bar plot representing the total peak area of the extracted components for the three nut samples under the different extraction conditions. Consistent with the fact that walnut is the nut with higher total polyphenolic content (3733 ± 1190 mg/100 g dry mass expressed as gallic acid equivalent, GAE) [26], it presents a larger quantity of extracted chemicals than almond and hazelnut. Regarding the extracting solvent composition, it was observed that the extraction capacity of pure organic solvents was not as effective as that achieved by pure water, except for the walnut sample. In contrast, mixtures of water with different amounts of organic solvents were quite effective, although a high percentage of it seemed to keep the extraction capacity constant or decrease it. Among the different extracting conditions evaluated, a great variety in extraction capacities was observed depending on both the extracting solvent employed and the nut sample matrix studied. For instance, acetone:water (50:50 and 70:30 *v*/*v*) provided the best extraction efficiencies for the walnut matrix; pure water and with mixtures of acetonitrile:water and acetone:water (both at 50:50 and 70:30 *v*/*v*) for hazelnut matrix; whereas for the almond matrix, the best extraction results were achieved when employing pure water and acetonitrile:water (80:20 *v*/*v*). Therefore, taking into consideration that the aim of the present work is to study the viability of a non-targeted HPLC-UV fingerprinting strategy to address nut sample classification, as a compromise, acetone:water (70:30 *v*/*v*) was chosen as the optimal extraction solvent for future experiments because of its satisfactory extraction efficiencies for the hazelnut and walnut samples.

### 3.2. Non-Targeted HPLC-UV Chromatographic Fingerprints

Once the extracting solvent was selected, the studied samples described in Table 1 were extracted, and their respective extracts were randomly analyzed, along with a QC and a water blank every ten sample injections, with the proposed reversed-phase HPLC-UV method.

**Figure 3:**
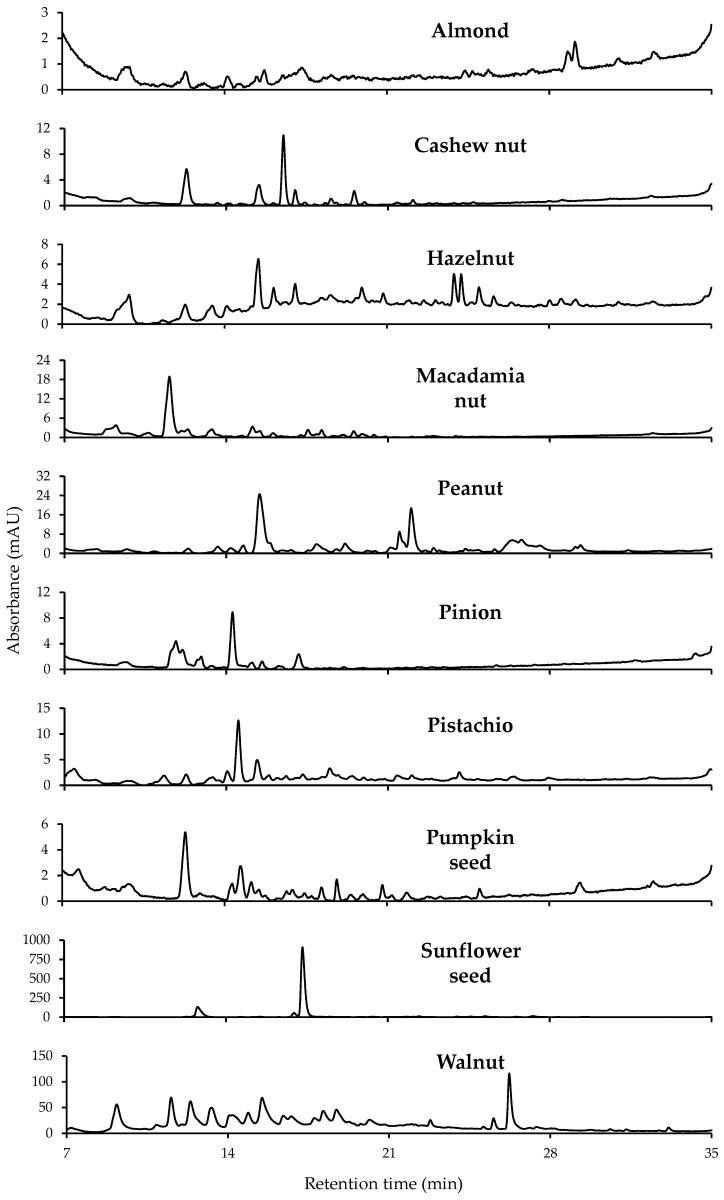
Non-targeted HPLC-UV chromatographic fingerprint acquired at 280 nm for a selected sample within each nut sample type. Chromatograms displayed only from minute 7 to 35 to remove absorption of the extracting solvent and column pre-conditioning step.

As an example, Figure 3 shows the chromatograms acquired at 280 nm for a selected sample within the ten types of nut samples under study. As can be seen, remarkable differences were obtained depending on the nut sample matrix analyzed, such as the number and distribution of extracted compounds or their signal intensity, with walnut and sunflower seed samples showing the highest ones. Therefore, due to these differences, the obtained fingerprints were proposed as possible chemical descriptors to achieve sample classification through the employment of chemometric methods.

### 3.3. Chemometric Data Analysis

#### 3.3.1. Characterization of Samples according to Nut Type: Non-Supervised PCA Study

The capability of the obtained non-targeted HPLC-UV chromatographic fingerprints to be used as discriminant chemical descriptors for sample classification and authentication depending on the nut fruit involved, independently of the presentation format (natural, fried or toasted), was evaluated by PCA. For that purpose, a 163 × 6001 dimension data matrix, which consisted of the absorbance signals recorded as a function of retention time for the 149 analyzed samples, as well as the QCs, was built. Moreover, autoscaling pretreatment was chosen to provide similar weight to all the variables (overall bioactive compound signals) as it suppresses differences in the magnitude and amplitude scale.

As a first result, Figure S1 (supplementary material) shows the obtained scores plot of PC1 vs. PC2, which does not present a compact group of the QCs as should be expected. In fact, when labelling them, a distribution based on their injection order is observed from the top-right section of the scores plot to the bottom-left section, which reveals a trend associated to the HPLC-UV sample sequence employed. Thus, this chromatographic behavior along the sequence affects systematically the fingerprinting signal registered for each sample, and therefore, the chemometric results displayed in this figure cannot be used, and a correction is required.

**Figure 4:**
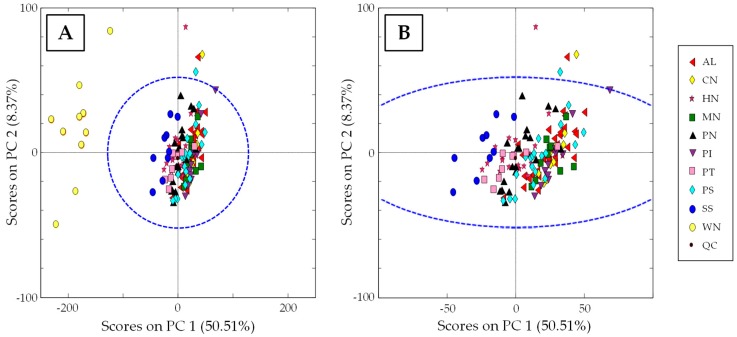
(**A**) PCA Score plot of PC1 vs. PC2 when using non-targeted HPLC-UV chromatographic fingerprints registered at 280 nm as chemical descriptors for all the analyzed samples. (**B**) Same PCA plot without including walnut samples.

As QCs are injections of the same extract, they not only allow the detection of any possible instrumental or chromatographic issue but also to correct them. In this case, the fingerprints obtained for each sample were normalized by dividing their absorbance signal variables by those of the closest QC in the sequence, whereas each QC was divided by itself. After performing this correction, QC samples appear in the same position in the PCA scores plot (see Figure 4A), and chemometric results can be discussed. As can be seen, walnut samples are clearly separated at the left part of the plot from the other nut samples (displaying negative PC1 score values). This difference could be related to the fact that they are among the matrices with higher polyphenolic content, as previously mentioned.

To better observe the distribution of the other samples, Figure 4B shows an extension of the PCA scores plot without including walnut samples. A trend along the PC1 can be seen that slightly groups the samples according to their nut type. Moreover, PC1 could be related to the total polyphenolic content as it seems to be a decrease in the level of extracted compounds in samples from the left to right part of the scores plot. For instance, sunflower seeds, showing the highest polyphenol content in dry mass (1400 ± 90 mg GAE/100 g) after walnut samples [26], are the group of samples located more to the left of the plot. Then, being distributed consecutively to the right of the plot following PC1, hazelnuts (550 ± 130 mg GAE/100 g dry mass) and pistachios (642 ± 5 0 mg/100 g dry mass), peanuts (460 ± 90 mg GAE/100 g dry mass), pumpkin seeds (140 ± 20 mg/100 g dry mass) [26], cashew nuts (133 mg GAE/100 g peeled dry mass) [27], macadamia nuts, almonds (58–159 mg GAE/100 g dry mass) [28,29], and pinions, can be found. Thus, even though there is not a clear separation between groups, those of higher polyphenolic content are distinguished from lower ones.

#### 3.3.2. Classification of Samples According to Nut Type: Supervised PLS-DA Study

**Figure 5:**
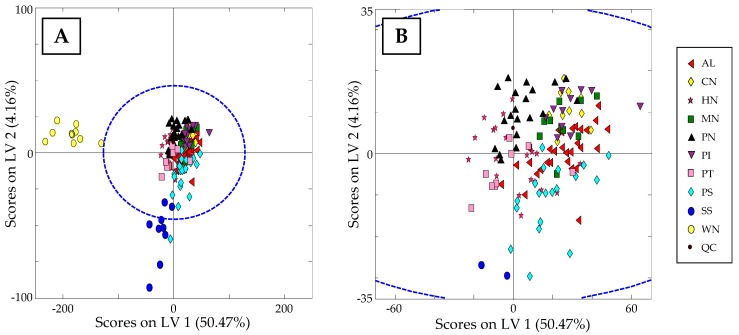
(**A**) Partial least squares-discriminant analysis (PLS-DA) Score plot of LV1 vs. LV2 when using non-targeted HPLC-UV chromatographic fingerprints registered at 280 nm as chemical descriptors for all the analyzed samples. (**B**) Same PLS-DA score plot without including walnuts and sunflower seeds samples.

HPLC-UV chromatographic fingerprints were also used as chemical descriptors to address nut classification by using a supervised PLS-DA method, obtaining the plot of scores of LV1 vs. LV2 depicted in Figure 5A. Similar to the reported results by PCA, the observed discrimination between samples could be associated to the total amount of extracted compounds, even though in this case both LV1 and LV2 are contributing to it. In fact, PLS-DA maximized the classification of the two nuts with the highest polyphenolic content (walnuts and sunflower seeds), whereas the other samples are more concentrated in the center of the graph. To focus on this distribution, in Figure 5B, walnut and sunflower seed samples are excluded. Again, some samples, such as hazelnuts, pistachios, and peanuts, in which a higher number of extracted phytochemicals is expected according to the literature, can be distinguished from the other samples mainly due to LV1.

The fact that the analyzed nut samples are not distributed randomly but more or less grouped according to the type of nut fruit, independently of the format of presentation, shows that non-targeted HPLC-UV fingerprints can be employed as adequate chemical descriptors to address nut sample classification and authentication.

**Figure 6:**
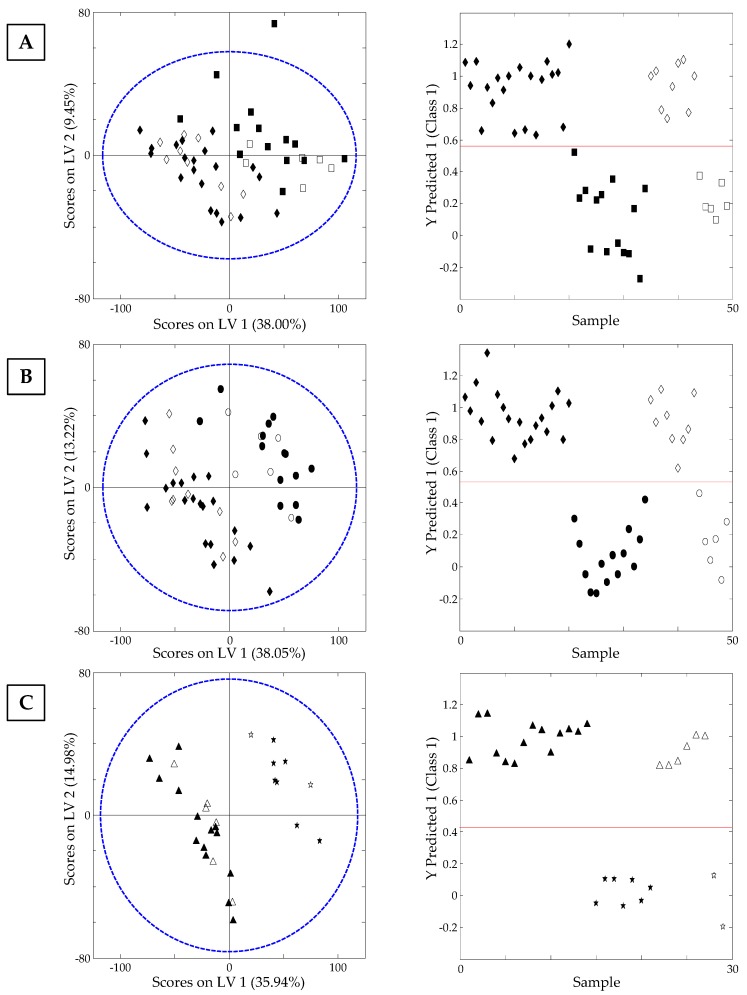
On the left, PLS-DA score plot projected in LV1 vs. LV2 and on the right, Sample vs. Y predicted 1 for (**A**) almonds ◆ vs. hazelnuts ▮, (**B**) almonds ◆ vs. peanuts ● and (**C**) pumpkin seeds ▲ vs. sunflower seeds ★. Filled and empty symbols correspond to calibration and validation sets, respectively.

As previously commented in the introduction section, the adulteration of high quality or expensive nut products by substituting them with a lower quality or cheaper nut is a common practice nowadays. For this reason, in the present work, PLS-DA models were built to study some nuts in pairs, i.e., almonds vs. hazelnuts, almonds vs. peanuts or pumpkin seeds vs. sunflower seeds. As can be seen in Figure S2 (supplementary material), the number of latent variables employed to generate each PLS-DA model was selected depending on the cross-validation classification error average, being approximately the first minimum point the most appropriate one. As a good classification was obtained for the studied pairs, the models were validated by using a 70% of each group of samples as the calibration set, while the remaining 30% of samples constituted the validation set. Figure 6 shows the obtained PLS-DA score plots projected on LV1 vs. LV2 as well as Samples vs. Y predicted 1 for (A) almonds vs. hazelnuts, (B) almonds vs. peanuts, and (C) pumpkin seeds vs. sunflower seeds, obtaining a perfect classification and discrimination between these nut samples and reaching a prediction rate of 100% in each case. Therefore, the proposed strategy based on the use of non-targeted HPLC-UV chromatographic fingerprints registered at 280 nm is a very promising method to achieve the characterization, classification, and authentication of nut samples, as well as to address the future identification of some nut frauds by means of adulteration with cheaper nut products.

#### 3.3.3. Classification of Samples According to Their Processing Thermal Treatment: Supervised PLS-DA Study

**Figure 7:**
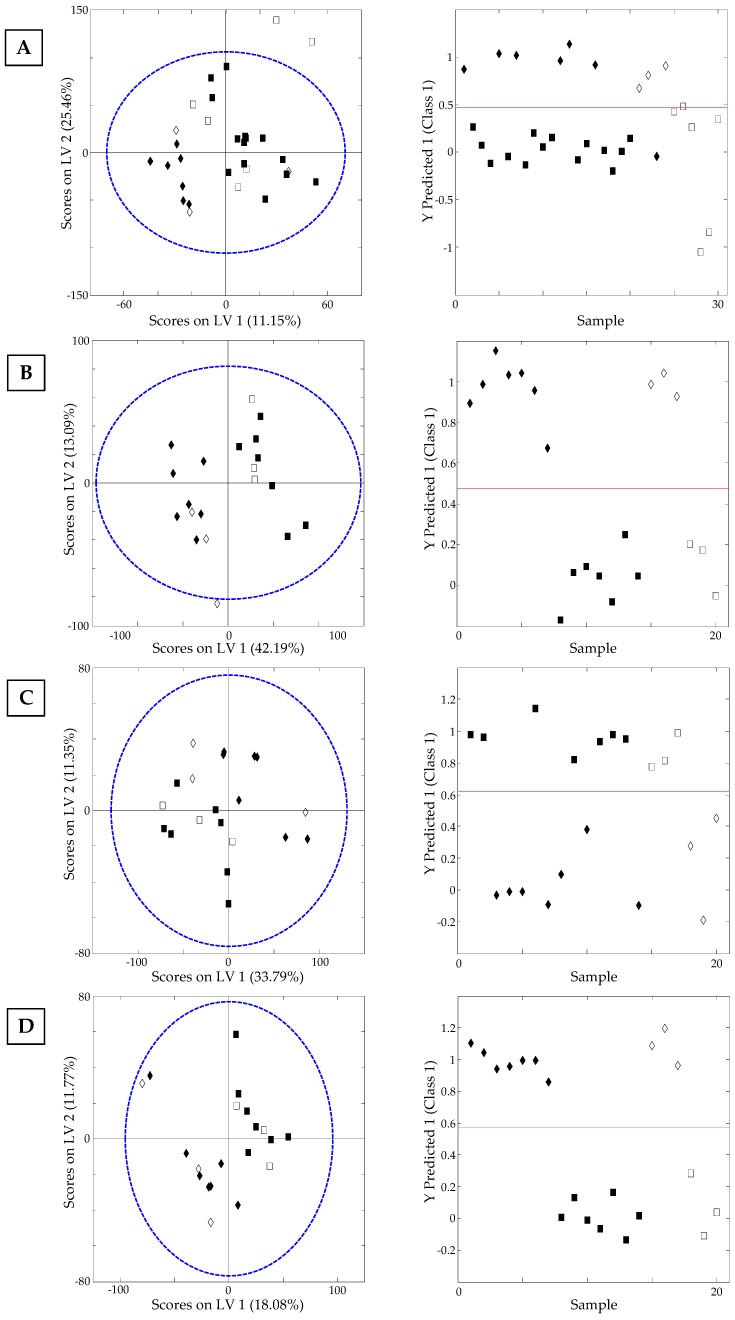
On the left, PLS-DA score plot projected in LV1 vs. LV2 and on the right, Sample vs. Y predicted 1 for (**A**) natural ◆ vs. thermally treated (fried or toasted) almonds ▮, (**B**) natural ◆ vs. toasted hazelnuts ▮, (**C**) fried ◆ vs. toasted peanuts ▮, and (**D**) toasted ▮ vs. natural pumpkin seeds ◆. Filled and empty symbols correspond to calibration and validation sets, respectively.

The applicability of non-targeted HPLC-UV chromatographic fingerprints as chemical descriptors to achieve nut sample classification regarding other nut food features, such as the nut format presentation according to the thermal processing treatment (natural and thermally processed, fried or toasted), was also evaluated. For that purpose, the chromatographic fingerprints of those nuts with different types of presentation (see Table 1) were used to create the data matrices, which were later subjected to supervised PLS-DA study. Thus, PLS-DA models were built for almonds, hazelnuts, peanuts, and pumpkin seeds following the same criterion for the number of latent variables selection as established in previous PLS-DA models, as can be seen in Figure S3 (supplementary material). A very acceptable discrimination of the analyzed samples according to the thermal processing method was achieved. Hence, 30% of the samples for each group were removed from the model and used as a validation set. As can be seen in Figure 7, where the PLS-DA score plots projected on LV1 vs. LV2, as well as Samples vs. Y predicted 1 for (A) almonds, (B) hazelnuts, (C) peanuts, and (D) pumpkin seeds, are represented, while for almonds the model showed a 78% classification rate between natural and thermally processed samples (toasted and fried), the other studied nut samples presented a value equal to 100%.

## 4. Conclusions

In this work, non-targeted HPLC-UV chromatographic fingerprints recorded at 280 nm have proved to be a useful and dependable tool for the classification and authentication of nuts, according to their nut type as well as their thermal treatment, when combined with chemometrics. In fact, the built PLS-DA models for the distinction of a determinate type of nut in front of another have reached a classification rate equal to 100%, independently of their thermal treatment. Moreover, supervised models have also allowed a discrimination capacity over 78% regarding the thermal processing treatment in each nut type. Therefore, this strategy could be proposed to detect frauds involving any of the nut samples studied.

## Figures and Tables

**Table 1:** Description of the nut samples analyzed.

Nut Type	Abbreviation	Number of Samples			Origin
		Natural	Fried	Toasted	
Almonds	AL	10	10	10	Spain-USA
Cashew nuts	CN	-	10	-	Brazil
Hazelnuts	HN	10	-	10	Spain-Turkey
Macadamia nuts	MN	10	-	-	South Africa
Peanuts	PN	-	10	10	Spain-Brazil-China-USA
Pinions	PI	10	-	-	Spain-China
Pistachios	PT	-	-	9	Spain-Germany-Iran
Pumpkin seeds	PS	-	10	10	Austria-China
Sunflower seeds	SS	-	-	9	Spain
Walnuts	WN	10	-	-	Chile-USA

## Supplementary Materials

The following are available online at https://www.mdpi.com/1424-8220/19/6/1388/s1, Figure S1: PCA Score plot of PC1 vs. PC2 employing non-targeted HPLC-UV chromatographic fingerprints registered at 280 nm, without QC correction, for all the nut samples and QCs analyzed, Figure S2: Latent variable number vs. CV classification error average plots for the built PLS-DA models of: (A) almonds vs. hazelnuts, (B) almonds vs. peanuts, and (C) pumpkin seeds vs. sunflower seeds, Figure S3: Latent variable number vs. CV classification error average plots for the built PLS-DA models of (A) almonds, (B) hazelnuts, (C) peanuts, and (D) pumpkin seeds.
