# Supplementary material for: Non-Targeted HPLC-UV Fingerprinting as Chemical Descriptors for the Classification and Authentication of Nuts by Multivariate Chemometric Methods
Source: Sensors (Basel). 2019 Mar 21;19(6):1388. doi: 10.3390/s19061388 (PMC6471388; doi:10.3390/s19061388)

Supplementary Material

Non-targeted HPLC-UV Fingerprinting as Chemical Descriptors for the Classification and Authentication of Nuts by Multivariate Chemometric Methods

Guillem Campmajó [1], Gemma J. Navarro [1], Nerea Núñez [1], Lluís Puignou [1,2], Javier Saurina [1,2] and Oscar Núñez [1,2,3,*]

[1] Department of Chemical Engineering and Analytical Chemistry, University of Barcelona, Martí i Franqués,
1-11, E08028 Barcelona, Spain; campma03@gmail.com (G.C.); g.navarro.albiol@gmail.com (G.J.N.); nereant7@gmail.com (N.N.), lluis.puignou@ub.edu (L.P.), xavi.saurina@ub.edu (J.S.)

[2] Research Institute in Food Nutrition and Food Safety, University of Barcelona, Recinte Torribera, Av. Prat de la Riba 171, Edifici de Recerca (Gaudí), Santa Coloma de Gramenet, E08921 Barcelona, Spain

[3] Serra Húnter Fellow, Generalitat de Catalunya, Rambla de Catalunya 19-21, E08007 Barcelona, Spain

* Correspondence: oscar.nunez@ub.edu; Tel.: +34-93-403-3706

Fig. S1. PCA Score plot of PC1 vs PC2 employing non-targeted HPLC-UV chromatographic fingerprints registered at 280 nm, without QC correction, for all the nut samples and QCs analyzed.

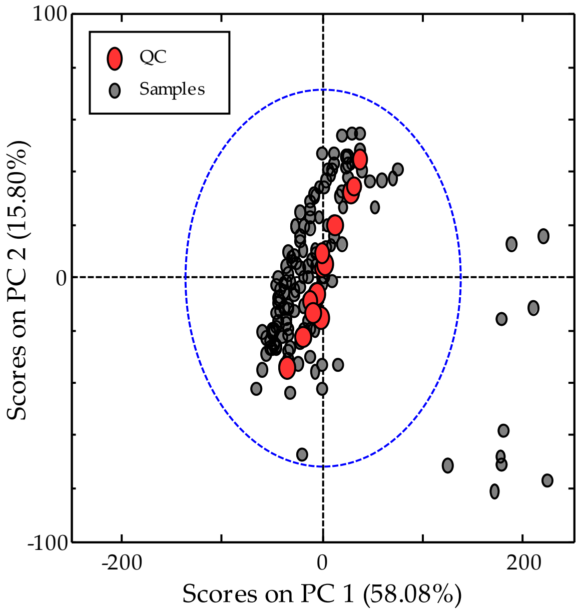

Fig. S2. Latent variable number *vs* CV classification error average plots for the built PLS-DA models of: (a) almonds *vs* hazelnuts, (b) almonds *vs* peanuts and (c) pumpkin seeds *vs* sunflower seeds.

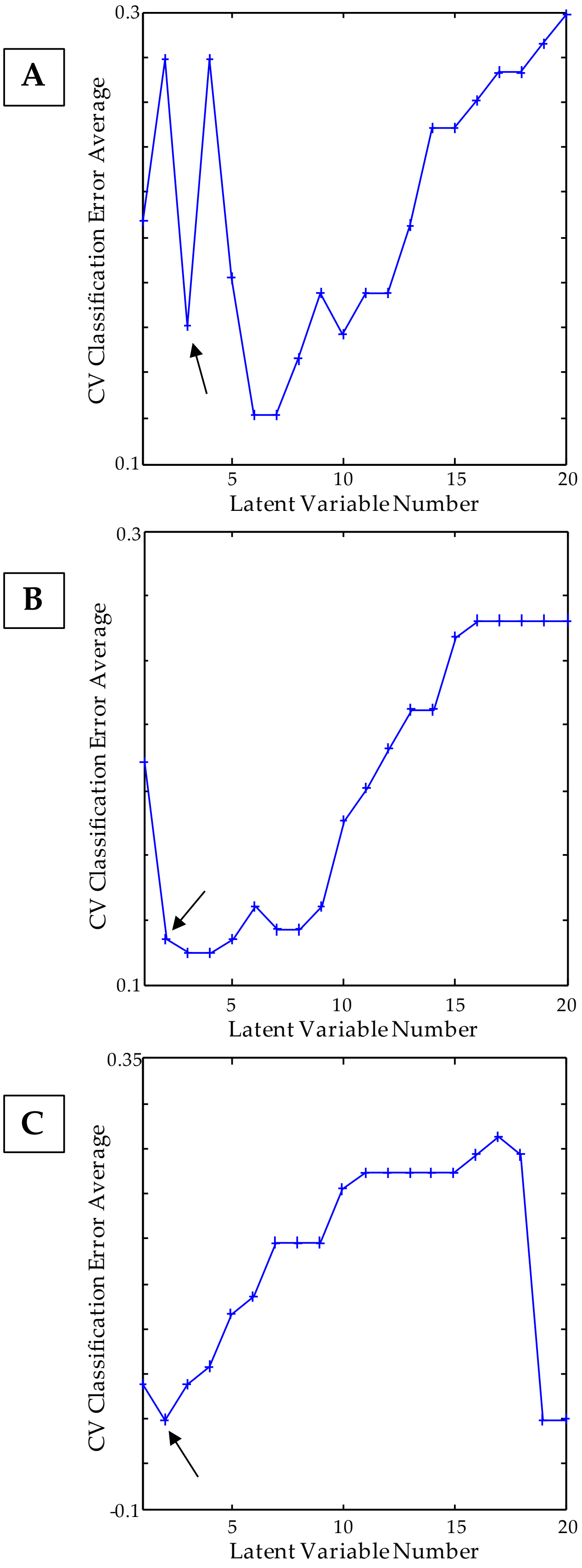

Fig. S3. Latent variable number *vs* CV classification error average plots for the built PLS-DA models of: (a) almonds, (b) hazelnuts, (c) peanuts and (d) pumpkin seeds.

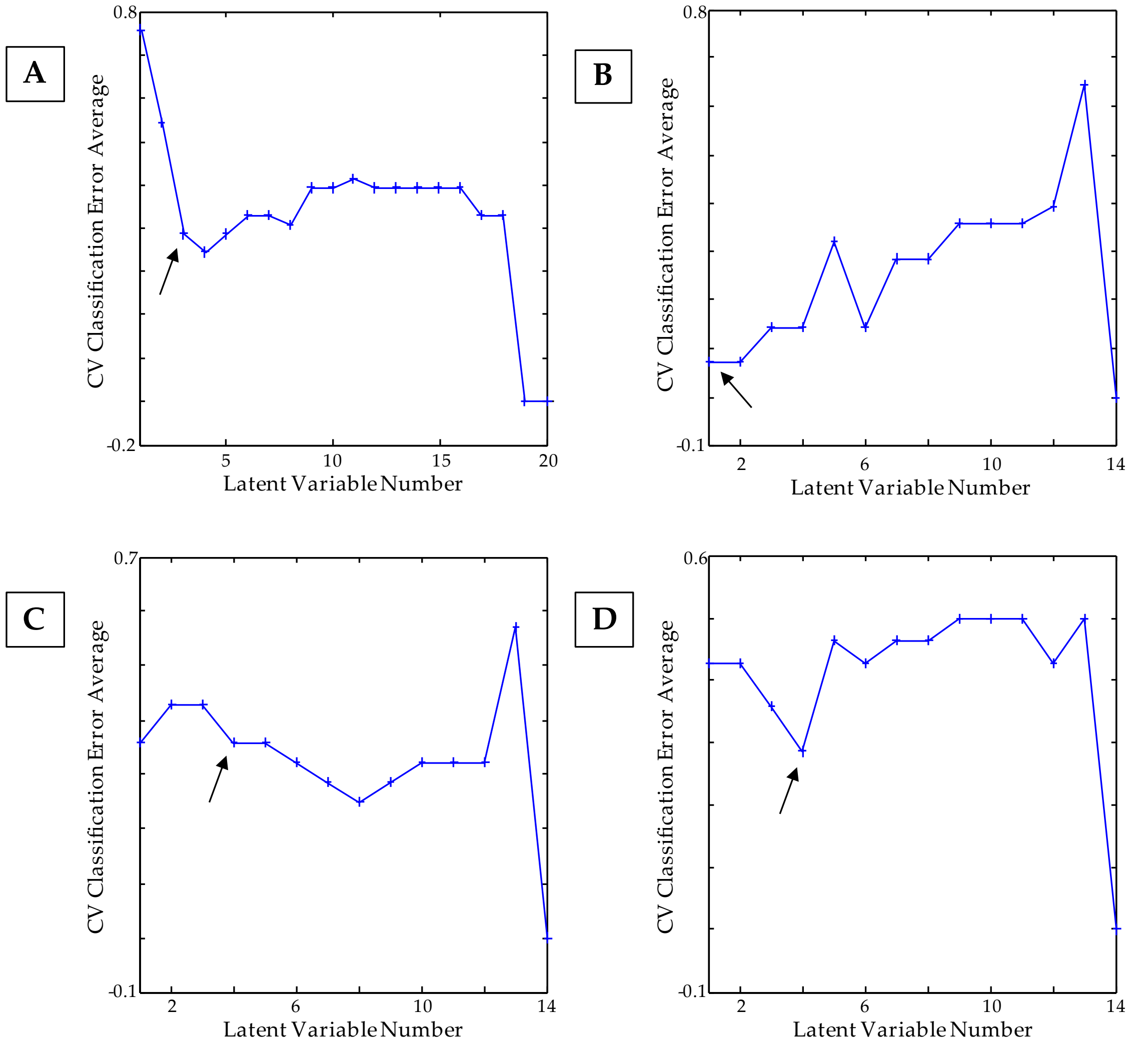

Supplement: Supplementary file 1 [file sensors-19-01388-s001.pdf]
